# A Study of the Safety and Morbidity Profile of Closed versus Open Technique of Laparoscopic Primary Peritoneal Access Port in Patients Undergoing Routine Laparoscopic Cholecystectomy at a Tertiary Care Hospital in Northeastern India

**DOI:** 10.1155/2022/1017551

**Published:** 2022-07-12

**Authors:** A. Baruah, N. Topno, S. Ghosh, N. Naku, R. Hajong, D. Tongper, D. Khongwar, P. Baruah, N. Chishi, S. Sutradhar

**Affiliations:** ^1^Department of Surgery, North Eastern Indira Gandhi Regional Institue of Health and Medical Sciences, Shillong, Meghalaya, India; ^2^Department of Surgical Oncology, Sri Aurobindo Medical College and PG Institute, Indore, M.P., India; ^3^Department of Biochemistry, North Eastern Indira Gandhi Regional Institue of Health and Medical Sciences, Shillong, Meghalaya, India; ^4^Department of Neurology & Neurosurgery, J.A Hospital Campus GRMC, Laskar, Gwalior, M.P., India

## Abstract

**Introduction:**

Laparoscopic cholecystectomy (LC) is the gold standard operation for gallstone disease. Primary port placement into the abdomen is a blind procedure and is challenging with chances of unforeseen complications. The complication rate has remained the same during the past 25 years. Both closed/Veress and open/Hasson's techniques are commonly employed and have their typical indications for use.

**Materials and Methods:**

This prospective study was carried out in the Department of General Surgery, North Eastern Indira Gandhi Regional Institute of Health and Medical Sciences (NEIGRIHMS), Shillong, from January 2014 to January 2016, with the aim to compare the safety profile of closed/Veress and open/Hasson's methods of access to the abdomen during laparoscopic cholecystectomy (LC). The study had 400 eligible cases undergoing LC who were randomly allotted into 2 groups with 200 cases each: group A: closed/Veress needle method and group B: open/Hasson's method.

**Results:**

Closed/Veress and open/Hasson's method of establishing pneumoperitoneum in laparoscopic cholecystectomy is equally safe in terms of major complications. The closed/Veress method gives faster access to the abdomen as compared to the open method (5.62 ± 2.23 minutes and 7.18 ± 2.52 minutes, respectively, *p* value <0.0001). The open/Hasson's method is associated with more primary port site complications (9/200 vs. 0/200, *p* value 0.0036) and troublesome intraoperative gas leaks (39/200 vs. 2/200, *p* value <0.0001). The open technique for primary peritoneal access port for laparoscopic cholecystectomy does not impart any additional benefits in terms of safety and morbidity profile in patients undergoing LC.

**Conclusion:**

The closed/Veress method of establishing pneumoperitoneum in laparoscopic cholecystectomy is equally safe in terms of major complications and gives quicker access to the abdomen as compared to the open method.

## 1. Introduction

Laparoscopic cholecystectomy (LC) is the gold standard operation for gallstone disease. Primary port placement into the abdomen through small incisions for insertion of laparoscopic surgical instruments which is a blind procedure is challenging and fraught with complications. Access is associated with injuries to the gastrointestinal tract structures and major blood vessels, and at least 50% of these major complications occur before commencement of the intended surgery [1, 2]. This complication rate has remained the same during the past 25 years.

## 2. Materials and Methods

This prospective study was carried out in the Department of General Surgery, North Eastern Indira Gandhi Regional Institute of Health and Medical Sciences (NEIGRIHMS), Shillong, from January 2014 to January 2016, with the aim of comparing the safety profile of open versus closed methods of access to the abdomen during LC.

A total of 400 patients admitted for LC were enrolled in the study after due informed consent from the patients. The study was approved by the institutional ethics committee. Single blinding was adopted where patients were unaware of the group to which they would be allocated. The study group of patients consisted of 129 males and 271 females and they were allotted randomly into 2 groups: group A using the closed/Veress needle method (200 patients) and group B using the open/Hasson's method (200 patients). LC was performed by surgeons having more than 5 years' experience in the field of laparoscopic surgery.

### 2.1. Inclusion Criteria

The inclusion criteria were as follows:

- All patients undergoing routine LC
- Patients above 18 years of age
- Diagnosed to be calculous cholecystitis on ultrasound

### 2.2. Exclusion Criteria

The exclusion criteria were as follows:

- Those unwilling to consent
- LC on pregnant women
- LC for indications other than calculous cholecystitis
- LC along with laparoscopic CBD exploration
- Previous abdominal operations

The personal details of the patient like name, age/gender, date of admission, date of operation, date of discharge, and complications were recorded in the proforma.

### 2.3. Closed/Veress Method

A transversely placed sub/supraumbilical stab skin incision of about 5-6 mm was employed, and then, subcutaneous tissue was bluntly dissected until fascia was palpable. The abdominal wall was lifted with one hand, while the Veress needle was held in the right hand like a dart and inserted through the fascia into the peritoneal cavity. The angle of Veress needle insertion varied from 45° in non-obese to 90° in obese [3]. Two clicks of the Veress needle were appreciated as it penetrated first the umbilical fascia and then the peritoneum. The confirmatory test for correct placement of Veress was to observe that the intraperitoneal pressure was below 8 mm Hg and gas was flowing freely.

After achieving adequate pneumoperitoneum with intraabdominal pressure of 10–12 mmHg, the Veress needle was replaced with the trocar and cannula. It was advanced in steady rotating manner until a hissing sound from the outer end of the cannula was heard or change in resistance was noticed.

### 2.4. Open/Hasson's Method

A 1.5–2 cm transverse or semicircular incision approximately was made in the inferior/superior umbilical fold, and the skin edges were retracted with small Langenbeck retractors and the fat separated from the umbilical scar [4–7]. The rectus sheath was picked up with an Allis forceps to facilitate lifting of the abdominal wall. A vertical incision was placed on the fascia and rectus sheath. Using good tissue retraction, the preperitoneal fat and the peritoneum were identified. The peritoneum was sequentially picked up using two Halstead's mosquito artery forceps and then incised with a pair of scissors. The little finger was then introduced through this incision to explore the area around the incision for any adhesions. The 10 mm cannula without the trocar was inserted through the incision. The cannula was fixed to the abdominal wall with a 1/0 silk suture to prevent leakage of the pneumoperitoneum.

Per operative findings like method of pneumoperitoneum creation and its duration, a number of attempts, incision size, port site bleeding, gas leak, and total gas used were recorded. Per operative complications like visceral or vascular injury and port site hematoma were noted. Postoperative complications like primary peritoneal access port site hematoma or infection were noted.

### 2.5. Statistical Analysis

The data were analysed by using INSTAT software (GraphPad Prism Software Inc, La Jolla, California. USA). The mean access time was calculated by Students *t*-test and the difference between the various complications among the group was calculated by Fisher's exact test. A *p* value of <0.05 was taken to be statistically significant.

## 3. Results

A total of 400 patients undergoing LC were randomly divided into 2 groups. Both groups were well matched c for age, sex, and body weight (Table 1).

Time taken for access in the 2 groups was calculated from skin incision to entry of first trocar. The difference in mean access time in group A (5.62 ± 2.23) versus group B (7.18 ± 2.52) was statistically significant (*p* < 0.0001) which meant that access time in the closed/Veress needle group was faster compared to the open/Hasson's method group. The majority (63.5%) of access in group A, i.e., the closed/Veress needle group was achieved in 1–5 minutes compared to group B, i.e., the open/Hasson's method group (72%) was achieved in 6–10 minutes (Table 2).

It was observed that intraoperative gas leak was a troublesome problem in the open/Hasson's method group (19.5%) compared to the closed/Veress method group (1%) and the difference was statistically significant (*p* < 0.0001) (Table 3). There were two omental injuries (non-expanding hematoma) in the group A (closed/Veress method) which were managed conservatively. There was one bowel injury (ileal serosal tear approximately 0.5 cm) in group B (open/Hasson's) detected on table and repaired laparoscopically using a single layer of seromuscular silk sutures. However, there were no major vascular injuries in either group. The difference between the incidences of omental injury and bowel injury between the two groups was not statistically significant (Table 3).

Primary port site superficial surgical site infections were observed only in the open group (4.5%) and were found to be statistically significant (*p*=0.0036). Port site hematoma was also observed only in the open/Hasson's group (2.5%), but there was no statistically significant difference with the closed/Veress needle group (Table 4).

## 4. Discussion

LC is the gold standard operation for gallstone disease. Abdominal access and creation of a pneumoperitoneum are the first important steps in any laparoscopic surgery and carry a potential risk of bowel and vascular injuries. These are unique to laparoscopic surgery and are rarely seen in open surgery [8]. Access is associated with injuries to the gastrointestinal tract and major blood vessels, and at least 50% of these major complications occur before commencement of the intended surgery [1, 2].

This complication rate has remained the same during the past 25 years, and hence, the technique of primary trocar entry in laparoscopy still remains a debatable topic. No single method is suitable for all cases of LC. The entry technique may be individualized in each case depending on the preoperative evaluation and surgical skill.

Today, the closed/Veress needle and open/Hasson's technique with their various modifications are the two widely used methods of primary abdominal access [9]. Hence, we compared these two methods in terms of access time, safety profile, and complications associated with each method.

We, in our study, found no instances of major vascular injuries in both the groups. However, the open/Hasson's group encountered one ileal injury compared to none in the closed/Veress group. Chapron et al. in their large series reported bowel and major vessel injury rates to be 0.04% and 0.01% in the closed technique (*n* = 8324) and 0.19% and 0% in the open technique (*n* = 1562), respectively, and had concluded that open laparoscopy does not reduce the risk of major complications during laparoscopic access [10]. In our study, there was no significant difference in major complications between the two groups. On the contrary, Taye et al. [11] in their comparative study of 3000 cases concluded that the open method was relatively a safer technique as far as major complications are concerned. Bathla et al. [12] had found that the open technique in primary trocar insertion is superior as the Veress method had caused small bowel perforation (2%); whereas, in our study, we had no small bowel injury using Veress needle.

We found intraoperative gas leak to be a statistically significant problem in the open/Hasson's method group (19.5%) compared to the closed/Veress method group (1%) which was similar to results reported by Juneja et al. [13] and Chotai et al. [14]. This complication was troublesome as it disturbed the tempo of the surgery with resultant longer operating time.

On analysis of the access time between the two groups, we found that using Veress needle access to the abdomen was significantly quicker as compared to the open/Hasson's method. Majority (63.5%) of access in the closed/Veress needle group was achieved in 1–5 minutes compared to only 28% in the open/Hasson's method group. Nawaz [15] in their study of 140 patients had found similar results with access time for creation of pneumoperitoneum and insertion of camera port with Veress needle (4 ± 1 min) was faster compared to the open group (5 ± 1 min). On the contrary, Chotai et al. [14] had found access time for creation of pneumoperitoneum and insertion of primary camera port was longer at 5.12 ± 2.51 minutes in the closed method versus 3.94 ± 2.7 minutes in the open method. Hamayun et al. [16] and Juneja et al. [13] also had found the open method to be faster compared to the Veress needle method.

Pawanindra et al. [17] reported 2.91% (22 cases) of periumbilical hematoma out of 755 cases of modified open port insertion. Nawaz [15] had found that 1.3% and 2.6% of their patients developed umbilical port site hematoma and umbilical port site infection compared to none in the Veress needle group. Akbar et al. [18] noted the incidence of wound infection was more in the open group but was not found to be statistically significant. We, in our study, found that 2.5% (5 of 200) of patients had port site hematoma and 4.5% (9 of 200) patients had port site infection in the open/Hasson's group compared to no such port site complication in the closed/Veress needle group. Port site infection occurring in the open/Hasson's group was statistically significant. These port site complications in the open/Hasson's group may be attributed to larger incisions, more tissue dissection, and trauma compared to its closed/Veress needle method counterparts.

## 5. Conclusions

The closed/Veress method of establishing pneumoperitoneum in laparoscopic cholecystectomy is equally safe in terms of major complications and gives quicker access to the abdomen as compared to the open/Hasson's method. The open/Hasson's method is associated with more port site complications and troublesome intraoperative gas leaks. Thus, the open technique for primary peritoneal access port for laparoscopic cholecystectomy does not impart any additional benefits in terms of safety and morbidity profile of patients.

## Figures and Tables

**Table 1:** Demographic profile of patients in two groups.

Variable	Group A (closed/Veress needle) (*n* = 200)	Group B (open/Hasson's) (*n* = 200)
Age (years)	36.21 ± 9.00	37.61 ± 8.75
Male	68	61
Female	132	139
Weight (Kg)	60.90 ± 11.21	69.12 ± 14.25

**Table 2:** Access time analysis in both groups.

Access time (in minutes)	Group A (closed/Veress needle) (*n* = 200)	Group B (open/Hasson's) (*n* = 200)	*P* value
1–5	127	56	
6–10	72	144	
>10	1	0	
Mean access time	5.62 ± 2.23	7.18 ± 2.52	<0.0001

**Table 3:** Complications in the two groups.

Complication	Group A (closed/Veress needle) (*n* = 200)	Group B (open/Hasson's) (*n* = 200)	*P* value
Intraoperative gas leak	2	39	<0.0001
Omental injury	2	0	0.4987
Major vascular injury	0	0	—
Bowel injury	0	1	1.00

**Table 4:** Port site complications.

Complications	Group A (closed/Veress needle) (*n* = 200)	Group B (open/Hasson's) (*n* = 200)	*P* value
Port site hematoma	0	5	0.0609
Port site SSI	0	9	0.0036
Port site hernia	0	0	—

SSI, surgical site infection; NB, port site SSI managed with wound care and antibiotics.

## Data Availability

The data used to support the findings of this study are available from the corresponding author upon request.
